# Supplementary material for: A CRISPR-del-based pipeline for complete gene knockout in human diploid cells
Source: J Cell Sci. 2023 Mar 7;136(6):jcs260000. doi: 10.1242/jcs.260000 (PMC10038147; doi:10.1242/jcs.260000)
Supplement: Supplementary information [file joces-136-260000-s1.pdf]

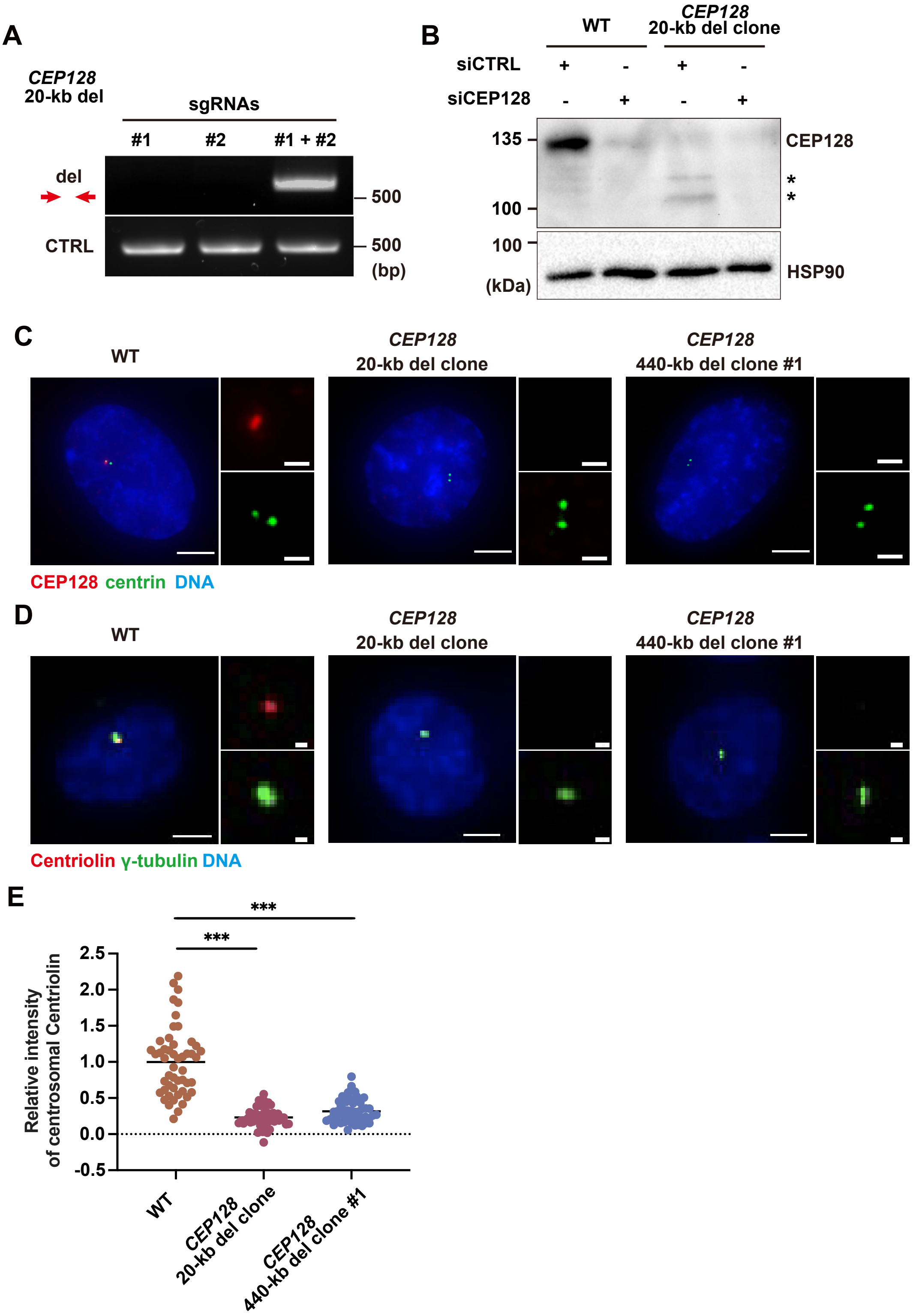

Journal of Cell Science • Supplementary information

Fig. S1. Validation of CEP128 deletion mutants.

A, Genomic PCR to detect the 20-kb deletion in cells electroporated with Cas9 and the indicated sgRNAs. **B**, Western blotting to analyze the protein expression of CEP128 in the cell lysate of WT and a *CEP128* 20-kb deleted clone at 72 hr after transfection of the indicated siRNA. Asterisks show smaller fragments of CEP128 protein. **C**, Immunofluorescence imaging of CEP128 and centrin in the cells of WT, the 20-kb or 440-kb *CEP128*-deleted clones. Scale bar: 5 μm (1 μm for insets). **D**, Immunofluorescence imaging of Centriolin and γ-tubulin in the cells of WT, the 20-kb or 440-kb *CEP128*-deleted clone. Scale bar: 5 μm (1 μm for insets). **E**, Quantification of relative Centriolin intensity at the centrosome from (**D**), 50 cells for each sample. Data are represented as mean and *P* value was calculated by Mann–Whitney U test. ***$P < 0.001$.

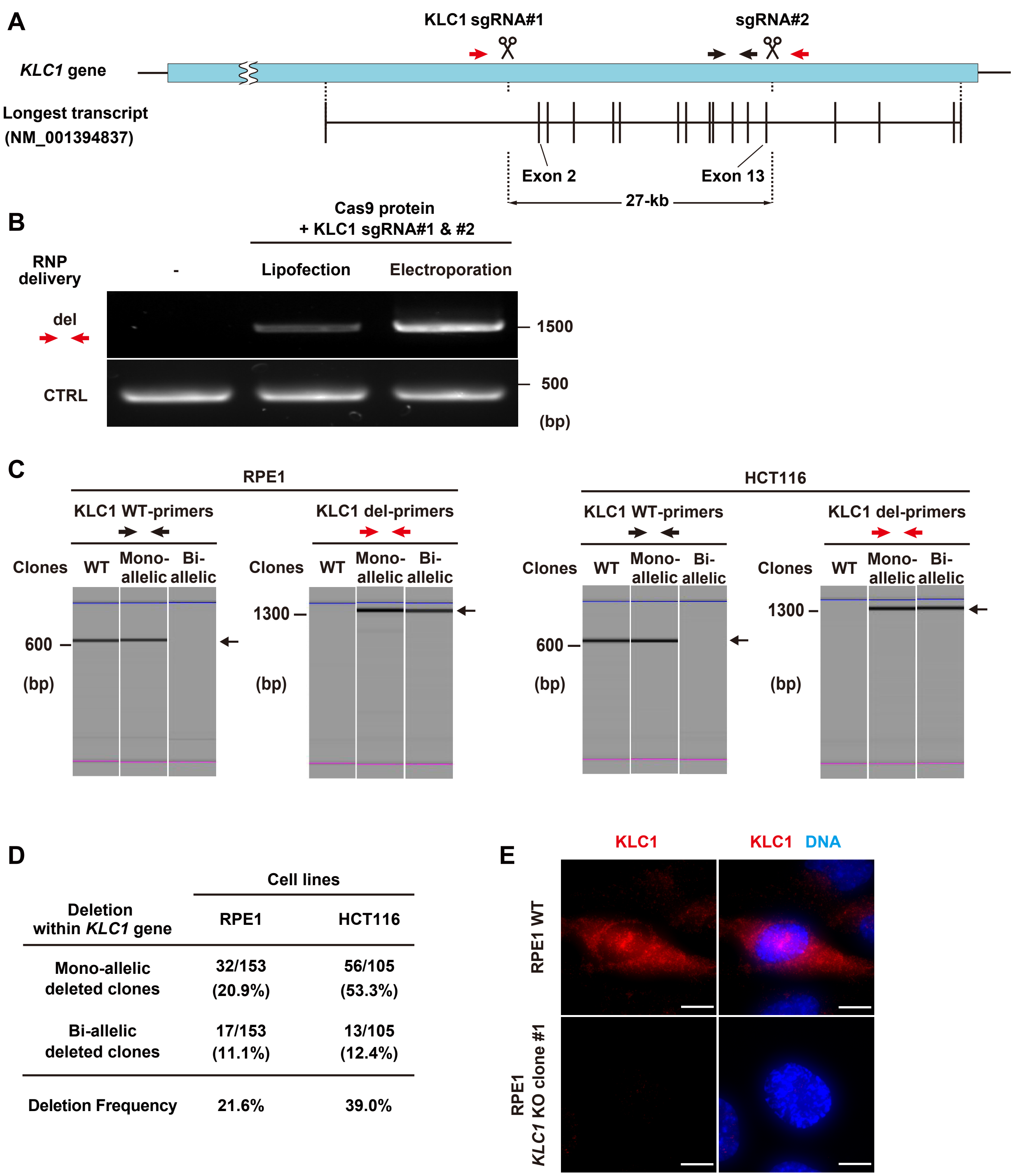

Deletion within *KLC1* gene	Cell lines: RPE1	HCT116
Mono-allelic deleted clones	32/153 (20.9%)	56/105 (53.3%)
Bi-allelic deleted clones	17/153 (11.1%)	13/105 (12.4%)
Deletion Frequency	21.6%	39.0%

Journal of Cell Science • Supplementary information

Fig. S2. A large chromosomal deletion within the *KLC1* gene in human diploid cells by the optimized CRISPR-del pipeline.

A, Schematic representation of *KLC1* gene and the longest transcript variant annotated in genome databases. The target positions of sgRNAs and the expected length of a large deletion are shown. Black and red arrows indicate primers to detect WT and the deleted region of *KLC1* gene, respectively. **B**, Genomic PCR to detect the 27-kb deletion in RPE1 cells electroporated or lipofected with Cas9 protein and the indicated sgRNAs using the indicated primers. **C**, Genomic PCR for detection of WT and the deleted alleles of *KLC1* gene in RPE1 and HCT116 cells, analyzed by the automated microchip electrophoresis system. Each electrophoresis pattern was adjusted according to the upper (blue) and lower (pink) size makers. The arrows on the right side of electrophoresis images indicate the specific PCR product. **D**, Summary for the efficiency of mono- and bi-allelic deletions within *KLC1* gene in RPE1 and HCT116 cells. **E**, Immunofluorescence imaging of KLC1 in WT and bi-allelic deletion mutant RPE1 cells. Scale bar: 5 µm.

Journal of Cell Science • Supplementary information

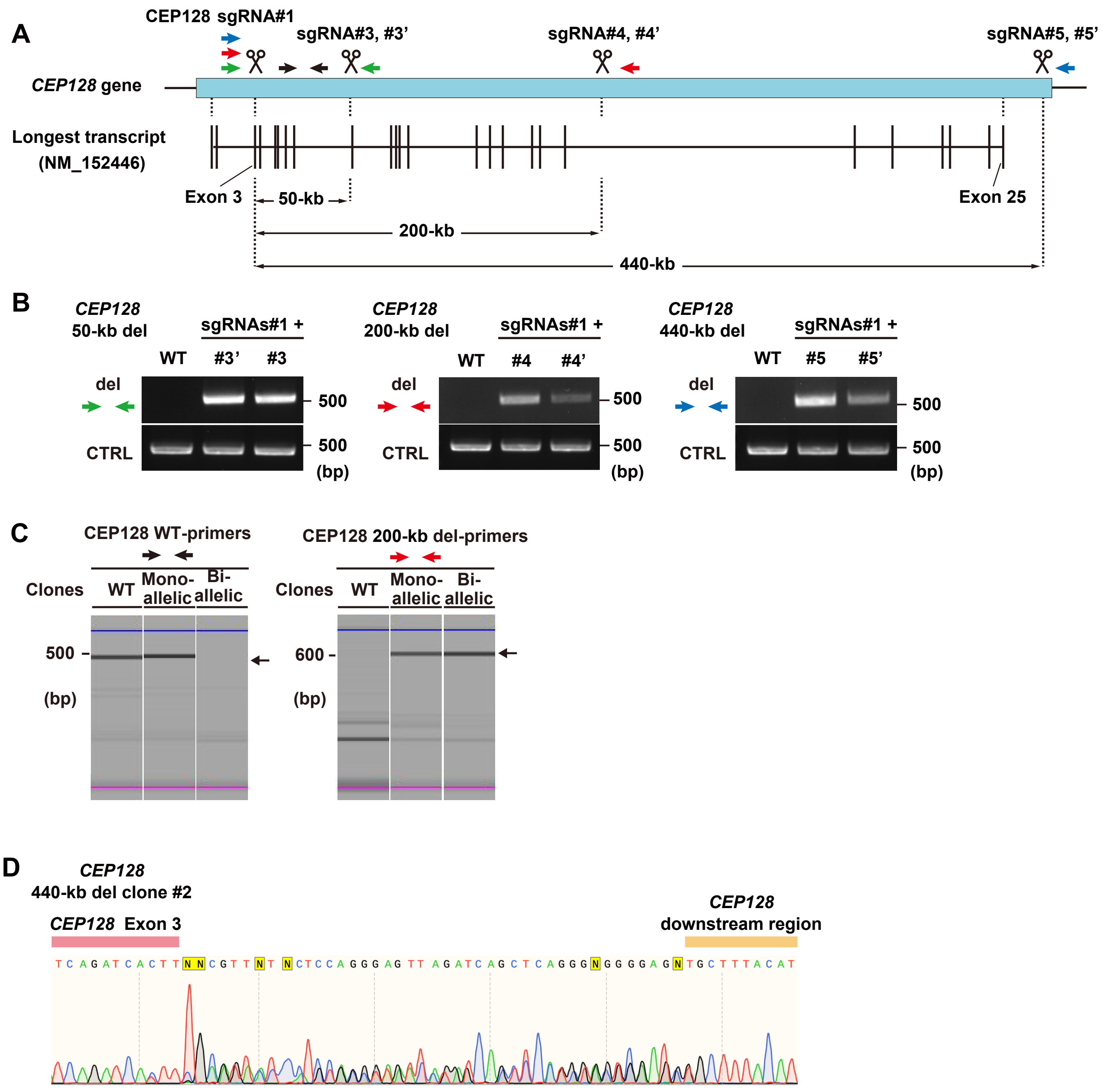

Journal of Cell Science • Supplementary information

Fig. S3. Large chromosomal deletions within the *CEP128* gene by the CRISPR-del pipeline.

A, Schematic representation of *CEP128* gene and the longest transcript variant annotated in genome databases. The target positions of sgRNAs and the expected lengths of large deletions are shown. Green, red, blue and black arrows indicate primers to detect the 50-kb, 200-kb and 440-kb deleted and WT regions, respectively. **B**, For the validation of sgRNA combinations, chromosomal deletions with different lengths within the *CEP128* gene were detected by genomic PCR. CEP128 sgRNA#3, #4 and #5, together with sgRNA#1, were used for further analyses in Fig. 2 and Fig. S2. **C**, Genomic PCR for detection of WT and the 200-kb deleted alleles of *CEP128* gene, analyzed by the automated microchip electrophoresis system. Each electrophoresis pattern was adjusted according to the upper (blue) and lower (pink) size makers. The arrows on the right side of electrophoresis images indicate the specific PCR product. **D**, Sequencing result of the *CEP128* deleted alleles in the 440-kb deleted clone #2.

Journal of Cell Science • Supplementary information

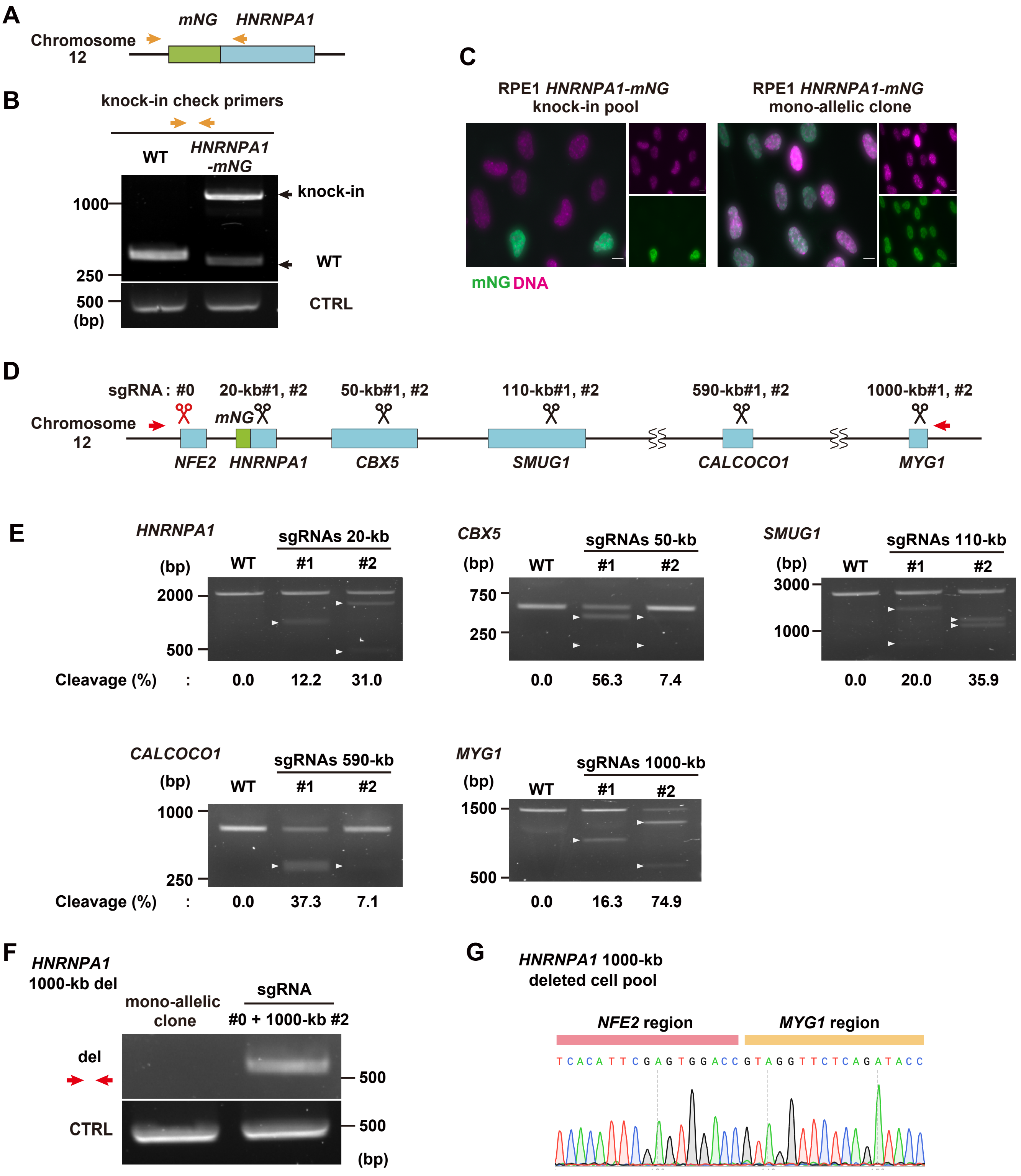

Journal of Cell Science • Supplementary information

Fig. S4. Large deletion of a chromosome region including *HNRNPA1* gene locus by the CRISPR-del.

A, Schematic representation of the *HNRNPA1* gene locus in RPE1 *HNRNPA1-mNG* cells. Orange arrows indicate the primers to detect the knock-in allele. B, Genomic PCR to detect WT and the knock-in alleles of the *HNRNPA1* gene in the indicated cells. **C**, Fluorescence imaging of HNRNPA1-mNG in the indicated cells. Scale bar: 10 µm. **D**, Schematic representation of the chromosome region around the *HNRNPA1-mNG* locus in RPE1 *HNRNPA1-mNG* cells. The target positions of sgRNAs are shown. Red arrows indicate the primers to detect the 1000-kb chromosomal deletion. **E**, The T7E1 assay to detect genome editing at the indicated gene loci in RPE1 WT cells electroporated with Cas9 protein and the indicated sgRNAs. **F**, Genomic PCR to detect the 1000-kb deletion in WT cells and cells electroporated with Cas9 protein and the indicated sgRNA pair. **G**, Sequencing result of the deletion band shown in (**F**).

Fig. 1E

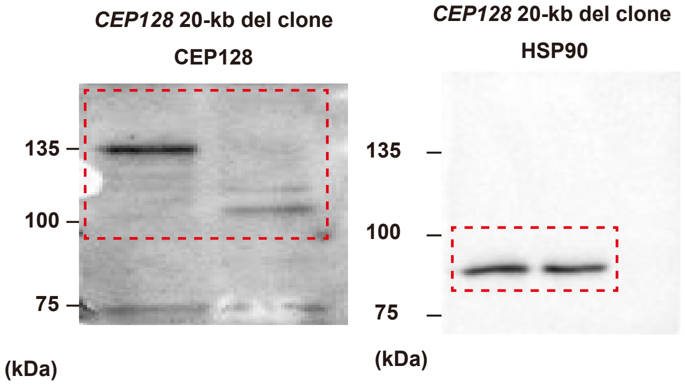

Fig. 2F

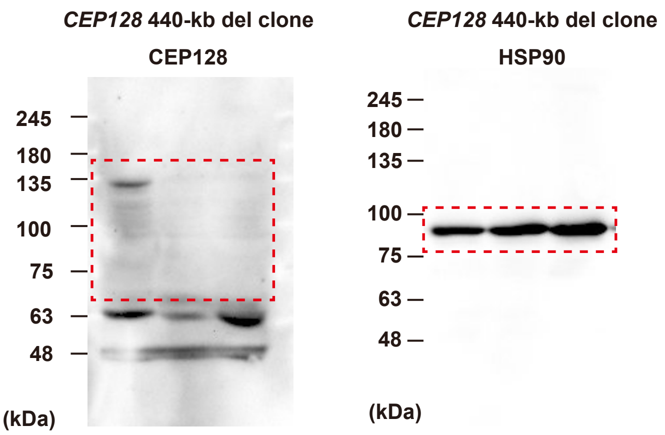

Fig. 4D

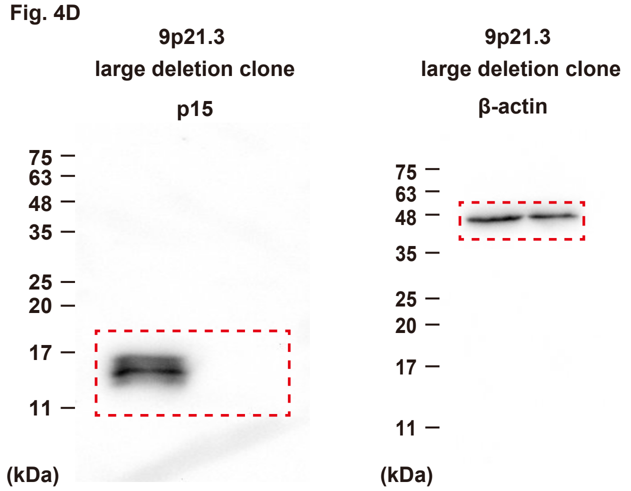

Fig. S1B

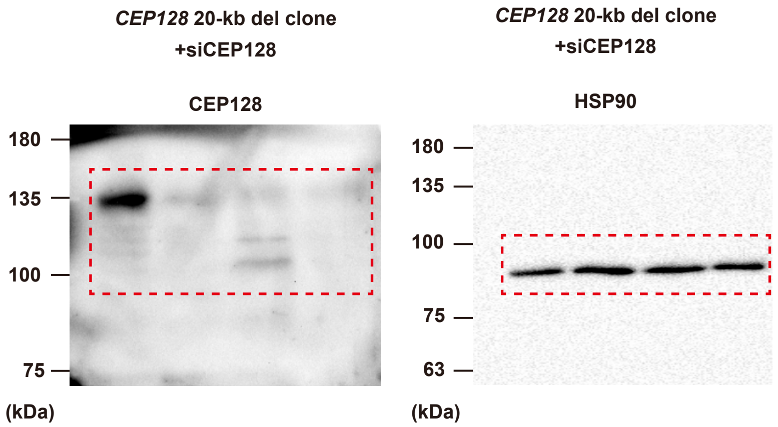

Fig. S5. Blot transparency.

Uncropped scans of western blots for Fig. 1E, 2F, 4D and S1B.

Journal of Cell Science • Supplementary information

Table S1. List of sgRNAs used in this study.

sgRNA/crRNA Target	Name	Sequence
CEP128 Exon3	CEP128 sgRNA#1	GACTCAATCGGTCACGACAG
CEP128 Intron8(20-kb)	CEP128 sgRNA#2	GATTTTTTCGGATGTACCAA
CEP128 Intron8(50-kb)	CEP128 sgRNA#3	CCTGACAAGGAACCTTATTG
CEP128 Intron19(200kb)	CEP128 sgRNA#4	TCAAGAAGGGCACTTTACGT
CEP128 downstream region(440-kb)	CEP128 sgRNA#5	GATGTATAGATAACACGGGG
KLC1 Intron1	KLC1 sgRNA#1	GAGGTGCTACTTGATAACAC
KLC1 Exon13	KLC1 sgRNA#2	CAACGTGGACGTGGTCAAGT
NFE2	HNRNPA1#0	TCACATTCGAGTGGACCATC
HNRNPA1	HNRNPA1 20-kb#1	ACGCTTTCAAGGAGGTGTAT
HNRNPA1	HNRNPA1 20-kb#2	GGATTGAGAGTGATCACTCA
CBX5	HNRNPA1 50-kb#1	ACCCAGGGAGCACAATACTT
CBX5	HNRNPA1 50-kb#2	TACCCAGGGAGCACAATACT
SMUG1	HNRNPA1 110-kb#1	GAAGTCTCTTATACCCACGG
SMUG1	HNRNPA1 110-kb#2	TGGGAACCATCCAATCCCT
CALCOCO1	HNRNPA1 590-kb#1	GTGGGAATAGAATCGTCCAC
CALCOCO1	HNRNPA1 590-kb#2	TGTGGGAATAGAATCGTCCA
MYG1	HNRNPA1 1000-kb#1	TGGCACCTTCCACTGCGACG
MYG1	HNRNPA1 1000-kb#2	GGTATCTGAGAACCTACCTC
MTAP	9p21.3 sgRNA#1	AAGTAAGCAGTTCTCCCACG
DMRTA1	9p21.3 sgRNA#2	TAGTGGATGTGGAGCCCAAA
HNRNPA1	crRNA HNRNPA1	ATTAGGTAAGTAAGCACCTT

Journal of Cell Science • Supplementary information

Table S2. List of primers used in this study.

Primer for PCR-assembled DNA templates	Name	Sequence
sgRNA amplification primers	Universal Design Primer_Fw	TTCTAATACGACTCACTATAG
	Universal Design Primer_Rv	AAAAGCACCGACTCGGTG
crRNA_tracrRNA	crRNA_tracrRNA	GTTTTAGAGCTAGAAATAGCAAGTTAAAATAAGGCTAGTCCGTTATCAACTTGAAAAAGTGGCACCGAGTCGGTGCTTTT
CEP128 sgRNA#1	sgRNA_CEP128_#1_Fw	TTCTAATACGACTCACTATAGACTCAATCGGTCACGACAG
	sgRNA_CEP128_#1_Rv	TTCTAGCTCTAAAACCTGTCGTGACCGATTGAGTC
CEP128 sgRNA#2	sgRNA_CEP128_#2_Fw	TTCTAATACGACTCACTATAGATTTTTTCGGATGTACCAA
	sgRNA_CEP128_#2_Rv	TTCTAGCTCTAAAACTTGGTACATCCGAAAAAATC
CEP128 sgRNA#3	sgRNA_CEP128_#3_Fw	TTCTAATACGACTCACTATAGCCTGACAAGGAACCTTATTG
	sgRNA_CEP128_#3_Rv	TTCTAGCTCTAAAACCAATAAGGTTCCTTGTCAGG
CEP128 sgRNA#4	sgRNA_CEP128_#4_Fw	TTCTAATACGACTCACTATAGTCAAGAAGGGCACTTTACGT
	sgRNA_CEP128_#4_Rv	TTCTAGCTCTAAAACACGTAAAGTGCCCTTCTTGA
CEP128 sgRNA#5	sgRNA_CEP128_#5_Fw	TTCTAATACGACTCACTATAGATGTATAGATAACACGGGG
	sgRNA_CEP128_#5_Rv	TTCTAGCTCTAAAACCCCCGTGTTATCTATACATC
KLC1 sgRNA#1	sgRNA_KLC1_#1_Fw	TTCTAATACGACTCACTATAGAGGTGCTACTTGATAACAC
	sgRNA_KLC1_#1_Rv	TTCTAGCTCTAAAACGTGTTATCAAGTAGCACCT
KLC1 sgRNA#2	sgRNA_KLC1_#2_Fw	TTCTAATACGACTCACTATAGACTTGACCACGTCCACGTTG
	sgRNA_KLC1_#2_Rv	TTCTAGCTCTAAAACCAACGTGGACGTGGTCAAGT
HNRNPA1#0	sgRNA_#0_Fw	TTCTAATACGACTCACTATAGTCACATTCGAGTGGACCATC
	sgRNA_#0_Rv	TTCTAGCTCTAAAACGATGGTCCACTCGAATGTGAC
HNRNPA1 20-kb#1	sgRNA_del20-kb_#1_Fw	TTCTAATACGACTCACTATAGACGCTTTCAAGGAGGTGTAT
	sgRNA_del20-kb_#1_Rv	TTCTAGCTCTAAAACATACACCTCCTTGAAAGCGTC
HNRNPA1 20-kb#2	sgRNA_del20-kb_#2_Fw	TTCTAATACGACTCACTATAGTGAGTGATCACTCTCAATCC
	sgRNA_del20-kb_#2_Rv	TTCTAGCTCTAAAACGGATTGAGAGTGATCACTCAC
HNRNPA1 50-kb#1	sgRNA_del50-kb_#1_Fw	TTCTAATACGACTCACTATAGACCCAGGGAGCACAATACTT
	sgRNA_del50-kb_#1_Rv	TTCTAGCTCTAAAACAAGTATTGTGCTCCCTGGGTC
HNRNPA1 50-kb#2	sgRNA_del50-kb_#2_Fw	TTCTAATACGACTCACTATAGTACCCAGGGAGCACAATACT
	sgRNA_del50-kb_#2_Rv	TTCTAGCTCTAAAACAGTATTGTGCTCCCTGGGTAC
HNRNPA1 110-kb#1	sgRNA_del110-kb_#1_Fw	TTCTAATACGACTCACTATAGAAGTCTCTTATACCCACGG
	sgRNA_del110-kb_#1_Rv	TTCTAGCTCTAAAACCCGTGGGTATAAGAGACTTC
HNRNPA1 110-kb#2	sgRNA_del110-kb#_2_Fw	TTCTAATACGACTCACTATAGTGGGAACCATCCAATCCCT
	sgRNA_del110-kb#_2_Rv	TTCTAGCTCTAAAACAGGGATTGGATGGTTCCCAC
HNRNPA1 590-kb#1	sgRNA_del590-kb_#1_Fw	TTCTAATACGACTCACTATAGTGGACGATTCTATTCCCAC
	sgRNA_del590-kb_#1_Rv	TTCTAGCTCTAAAACGTGGGAATAGAATCGTCCAC
HNRNPA1 590-kb#2	sgRNA_del590-kb_#2_Fw	TTCTAATACGACTCACTATAGTGGACGATTCTATTCCCACA
	sgRNA_del590-kb_#2_Rv	TTCTAGCTCTAAAACTGTGGGAATAGAATCGTCCAC
HNRNPA1 1000-kb#1	sgRNA_del1000-kb_#1_Fw	TTCTAATACGACTCACTATAGTGGCACCTTCCACTGCGACG
	sgRNA_del1000-kb_#1_Rv	TTCTAGCTCTAAAACCGTCGCAGTGGAAGGTGCCAC
HNRNPA1 1000-kb#2	sgRNA_del1000-kb_#2_Fw	TTCTAATACGACTCACTATAGGTATCTGAGAACCTACCTC
	sgRNA_del1000-kb_#2_Rv	TTCTAGCTCTAAAACGAGGTAGGTTCTCAGATACC
9p21.3 sgRNA#1	sgRNA_9p21.3_#1_Fw	TTCTAATACGACTCACTATAGAAGTAAGCAGTTCTCCCACG
	sgRNA_9p21.3_#1_Rv	TTCTAGCTCTAAAACCGTGGGAGAACTGCTTACTTC
9p21.3 sgRNA#2	sgRNA_9p21.3_#2_Fw	TTCTAATACGACTCACTATAGTAGTGGATGTGGAGCCCAAA
	sgRNA_9p21.3_#2_Rv	GTAGTGGATGTGGAGCCCAAAGTTTTAGAGCTAGAA
HNRNPA1	HNRNPA1_crRNA_Fw	TTCTAATACGACTCACTATAGTAATTTCTACTCTTGTAGAT
	HNRNPA1_crRNA_Rv	AAGGTGCTTACTTACCTAATATCTACAAGAGTAGAAATTAC

dsDNA repair template	Name	Sequence
HNRNPA1	HNRNPA1_dsDNA repair template_Fw	CACTTTGAAACTTTAAAAGAAAAATTGTACTTTTCAGGTGGCTATGGCGGTTCCAGCAGCAGCAGTAGCTATGGCAGTGGCAGAAGAT<br>TTGGAGCTGGTGCAGGTGCAG
	HNRNPA1_dsDNA repair template_Rv	ACTGCAATTATAATGTTAACTATGTTGCACTGCTCAGCTACATTAGGGTTATTGGGTTCATCAGCAATTTAAAAAATTATGTCAACACAC<br>AAAAAGGTGCTTACTTACCTAACTACTTGTACAGCTCGTCCATGC

Primer for deletion check	Name	Sequence
CEP128 del-primers_20-kb	del-primer_CEP128_20-kb_Fw	AATGTTCTTAATGGGACCGTCT
	del-primer_CEP128_20-kb_Rv	GGGAGAATTACATGGGGAAGTGA
CEP128 del-primers_50-kb	del-primer_CEP128_50-kb_Fw	TGGCAGAATCATCCAGCGAATC
	del-primer_CEP128_50-kb_Rv	ATTATCCATGAGCATGACCTGCC
CEP128 del-primers_200-kb	del-primer_CEP128_200-kb_Fw	TGGCAGAATCATCCAGCGAA
	del-primer_CEP128_200-kb_Rv	GGAGATCCAAGGCAAGCACT
CEP128 del-primers_440-kb	del-primer_CEP128_440-kb_Fw	GCAGAATCATCCAGCGAATCAG
	del-primer_CEP128_440-kb_Rv	TGTTGGCATTCCTATCACGTC
CEP128 WT-primers 20-kb	WT-primer_CEP128_20_Fw	TTTCTTCGCATGAGTGGTGC
	WT-primer_CEP128_20_Rv	TGGCACTGGGTCAGGTAAAC
CEP128 WT-primers 50~440-kb	WT-primer_CEP128_50-440_Fw	CCACGTGACAAGAGAACCCA
	WT-primer_CEP128_50-440_Rv	TGAGATGCCCTTCGTGCAAT
KLC1 WT-primers	WT-primer_KLC1_Fw	TCGGCCATCTGCTTATTGCT
	WT-primer_KLC1_Rv	GTTCCCATGGGCCGGTTAAG
KLC1 del-primers	del-primer_KLC2_Fw	CTGGCTTTGCAGTGATGAGC
	del-primer_KLC2_Rv	AGCATGCAGATACGACAGCA
NEK2 WT-primers	WT-primer_NEK2_Fw	TGGGCTTTTAATCCTGTGTGTGA
	WT-primer_NEK2_Rv	ACTCCCATCAACTAAGAGACCAAC
HNRNPA1 del-primers 1000-kb	del-primer_HNRNPA1_1000-kb_Fw	CCCTAAACGTCCAACTGCCT
	del-primer_HNRNPA1_1000-kb_Rv	AACTGTCACCAACTGCCACA
MTAP_outside WT-primers	WT-primer_MTAP-outside_Fw	AGGTAGAGCCAGACTGGGAG
	WT-primer_MTAP-outside_Rv	TGCAGACCTTTCCGGTTCTC
MTAP WT-primers	WT-primer_MTAP_Fw	GGAGGCTTGTCTGCAGTAGG
	WT-primer_MTAP_Rv	AGCTTTACAAGTCCGGTGGG
CDKN2A WT-primers	WT-primer_CDKN2A_Fw	AGAATTCTCCCCCGTCCGTA
	WT-primer_CDKN2A_Rv	CGTTTCCTTCCTCCGCGATA
CDKN2B WT-primers	WT-primer_CDKN2B_Fw	CACTGAGGAGAGATTCCCGC
	WT-primer_CDKN2B_Rv	GACATCCCACGAGCCATCAT
DMRTA1 WT-primers	WT-primer_DMRTA1_Fw	AGACTTGACTGCGACCAAGG
	WT-primer_DMRTA1_Rv	GCATAATCCAAAGCTGGCCG
DMRTA1_outside WT-primers	WT-primer_DMRTA1-outsideFw	CCGAATCAAAGCATGGGCAC
	WT-primer_DMRTA1-outsideRv	ACACCCGAATCCCTAAGCAA
9p21.3 del-primers	9p21.3_del-primerFw	AGAGGGTACGCTTGCAAATGA
	9p21.3_del-primerRv	AAAACACGTGGCTCGCCTA

Primer for knock-in check	Name	Sequence
HNRNPA1 Knock-in check primers	KI-primer_HNRNPA1_Fw	AAGAGTGTCTGTAGCTACTGCTGG
	KI-primer_HNRNPA1_Rv	ACTATGTTGCACTGCTCAGCTA

Primer for surveyor assay	Name	Sequence
Surveyor 20-kb	Surveyor-20kb-Fw	CGCTTGCGATTTCCTAGCAC
	Surveyor-20kb-Rv	CTGGCTAATGCCGCTCCATA
Surveyor 50-kb	Surveyor-50kb-Fw	TCCTTTTTGTCCGAATGTCCTCT
	Surveyor-50kb-Rv	GAGAAGGCAGAGGAGTGGTTT
Surveyor 110-kb	Surveyor-110kb-Fw	TGGGCTTCATGAGGTGGATG
	Surveyor-110kb-Rv	GCATCGCCCTTCCAAAAGTT
Surveyor 590-kb	Surveyor-590kb-Fw	CCCACATGGAAGGAGTGTCC
	Surveyor-590kb-Rv	TGTGAGGAGATGCGAGGAGA
Surveyor 100-kb	Surveyor-1000kb-Fw	TAGTCGGTCGCCTGTCCTTA
	Surveyor-1000kb-Rv	ACTGTCACCAACTGCCACAA
