# Supplementary material for: A CRISPR-del-based pipeline for complete gene knockout in human diploid cells
Source: J Cell Sci. 2023 Mar 7;136(6):jcs260000. doi: 10.1242/jcs.260000 (PMC10038147; doi:10.1242/jcs.260000)
Supplement: Supplementary file 1 [file joces-136-260000_review_history.pdf]

A CRISPR-del-based pipeline for complete gene knockout in human diploid cells

Takuma Komori, Shoji Hata, Akira Mabuchi, Mariya Genova, Tomoki Harada, Masamitsu Fukuyama, Takumi Chinen and Daiju Kitagawa

DOI: 10.1242/jcs.260000

Editor: David Glover

Review timeline

Original submission:	7 March 2022
Editorial decision:	8 June 2022
First revision received:	2 December 2022
Accepted:	2 February 2023

Original submission

First decision letter

MS ID#: JOCES/2022/260000

MS TITLE: A CRISPR-del-based pipeline for complete gene knockout in human diploid cells

AUTHORS: Takuma Komori, Shoji Hata, Akira Mabuchi, Mariya Genova, Tomoki Harada, Masamitsu Fukuyama, Takumi Chinen, and Daiju Kitagawa
ARTICLE TYPE: Tools and Resources

We have now reached a decision on the above manuscript.

To see the reviewers' reports and a copy of this decision letter, please go to: https://submit-jcs.biologists.org and click on the 'Manuscripts with Decisions' queue in the Author Area. (Corresponding author only has access to reviews.)

As you will see, their is a wide spectrum of responses from the reviewers. The reviewers do, however, raise a number of substantial criticisms that prevent me from accepting the paper at this stage. They suggest, however, that a revised version might prove acceptable, if you can address their concerns. If you think that you can deal satisfactorily with the criticisms on revision, I would be pleased to see a revised manuscript. We would then return it to the reviewers.

Please ensure that you clearly highlight all changes made in the revised manuscript. Please avoid using 'Tracked changes' in Word files as these are lost in PDF conversion.

I should be grateful if you would also provide a point-by-point response detailing how you have dealt with the points raised by the reviewers in the 'Response to Reviewers' box. Please attend to all of the reviewers' comments. If you do not agree with any of their criticisms or suggestions please explain clearly why this is so.

Reviewer 1

Advance summary and potential significance to field

© 2023. Published by The Company of Biologists under the terms of the Creative Commons Attribution License (https://creativecommons.org/licenses/by/4.0/).

Komori, Kitagawa and colleagues here present a study on CRISPR-mediated genome deletions in a series of loci. They describe an experimental pipeline for handling and screening a large number of candidate clones and show that their approach allowed the deletion of large genomic regions from the CEP128 locus, around the HNRNPA1 locus and from 9p21.3.

This study represents an optimization of a well-established approach to genome editing (using 2 well-separated guides), so that there is not a clear demonstration of a 'new tool or technique, or a sufficiently substantial advance of an existing tool…'. While the relatively high deletion efficiencies achieved will be of use for researchers wishing to use such approaches, there is limited novel biological insight derived from this work. Furthermore, there are some technical issues that reduce the clarity of the outcomes. On this basis, the submission does not appear suitable as a 'Tools and Resources' article in JCS.

Comments for the author

Major issues
1. The data in Figure 3 contrast with those in Figure 2, in that a 1000 kb deletion was achieved with an efficiency similar to that seen with a 100 kb (albeit with a low frequency for the 590 kb deletion). If this is to be ascribed to the individual gRNAs, a direct demonstration of different cutting efficiency should be provided. Otherwise, it is not clear what can be concluded, beyond the basic feasibility of large deletions using the authors' protocol.

2. The use of a pool in the HNRNPA1 experiment is problematic for several reasons. First, the binary readout means that the deletion of one tagged allele in a bi-allelically labelled cell will not be detected by the FACS assay. This issue may confound the quantitation. Second, the relative percentage of cells with a mono- and bi-allelic mNG tag may vary between experiments, which may also impact on the outcomes. Third, a putative mNG-negative population may proliferate differently after transfection, possibly leading to other confounding factors. These experiments should be repeated with a clonal starting population.

3. With respect to the HNRNPA1 locus experiment, a further issue is whether the disruption of any of the protein coding genes in which the guides are sited may be toxic to cells, individually or in combination, and thus affect the outcome of the editing experiments. The 2.4% of mNG negative cells with the 1000 kb deletion into the MYG1 locus might argue against that, but information about the genetic interactions between the other loci is limited. The authors should be cautious about this aspect.

4. Was a percentage editing determined for the experiment in Fig. 1? It would be useful to extend the curve information in Figure 2c, if this could be included.

5. The PCR experiments in Figs. 1d, 2d, 4b, 4c, S1a, S2b and S3f should include a positive control.

6. Details of the RPE1 cell line used in this study and its source should be provided.

Minor points
7. In terms of individual clone handling, how are individual/ selected clones of interest woken up from the 96 well dish?

8. With a binary outcome (mNG positive/ negative), it is not helpful to present the data in Figure 3 as stacked columns. A bar graph should indicate only one of the values and the y-axis could be adjusted to extend only to 30%, which would be much clearer.

Reviewer 2

Advance summary and potential significance to field

In this manuscript, Kitagawa and his colleagues reported an improved CRISPR-del approach to generate complete gene knockout cells. As the authors pointed out CRISPR/Cas9-mediated gene disruptions with a small indel do not always guarantee absence of the gene products, leaving so-called 'zombie protein'. Therefore it is better to remove most of the structural genes to ensure

© 2023. Published by The Company of Biologists under the terms of the Creative Commons Attribution License (https://creativecommons.org/licenses/by/4.0/).

complete knockouts. There have been reports in which a large DNA fragment was removed by cutting two different target sites with the CRISPR/Cas9 method. In this manuscript, the authors report that the deletion efficiency was greatly improved, compared to the previous reports. If total deletion mutant cell lines were generated with a high deletion efficiency, it would be helpful investigating specific gene functions with little concerns on experimental errors.

Comments for the author

In this manuscript, Kitagawa and his colleagues reported an improved CRISPR-del approach to generate complete gene knockout cells. As the authors pointed out, CRISPR/Cas9-mediated gene disruptions with a small indel do not always guarantee absence of the gene products, leaving so-called ‘zombie protein’. Therefore, it is better to remove most of the structural genes to ensure complete knockouts. There have been reports in which a large DNA fragment was removed by cutting two different target sites with the CRISPR/Cas9 method. In this manuscript, the authors report that the deletion efficiency was greatly improved, compared to the previous reports. If total deletion mutant cell lines were generated with a high deletion efficiency, it would be helpful investigating specific gene functions with little concerns on experimental errors.
They made six or seven points which are critical for generation of complete gene knockout cells with high efficiency. However, it would not be easy for an average laboratory to install all the points indicated in the manuscript. Some points should be more critical for deletion efficiency than the others. Therefore, this reviewer suggests to perform control experiments to determine the following points.

- The authors used RPE1 cells. They may try additional cell lines to make sure that their method also guarantees a high deletion efficiency in diverse cell types.
- It would be useful if the authors compare transfection efficiency of electroporation and other transfection methods.
- The authors should explain how to determine target sequences of a specific gene.
- The bi-allelic deletions of *CEP128* were much less efficient than the expectation from the mono-allelic deletions. The authors interpreted that the bi-allelic knockout of *CEP128* reduces the proliferative ability of the cells. This reviewer suggests to perform the same experiment with another genes whose deletions do not affect cell survival and proliferation.

<u>Reviewer 3</u>

Advance summary and potential significance to field

Komori et al. hand in a manuscript on a CRISPR/Cas9 method to generate large gene deletions in non-transformed human RPE-1 cells using two separate guide RNAs at a distance. In a nutshell, they combine in vitro transcription of guide RNAs to form RNPs with Cas9 that are delivered to cells by electroporation.
Single cell dilution derived clones are directly checked by genome PCR, which easily differentiates between wt and ko alleles due to the large deletions. Taking the CEP128 wild-type gene and recombinant fluorescently labeled (NeonGreen, mNG) hnRNPA1 as proofs of principle, the authors provide further evidence for the efficiency of the method. From deleting initially 20 kb around the CEP128 and hnRNPA1-mNG genomic sequences, they show that an increase up the 440 kb (CEP128) or even 1000 kB (hnRNPA1 and neighboring genes) is still possible albeit reduced frequency of monoallelic and biallelic deletion.
Finally, the authors successfully delete of a large region in in 9p21.3 comprising several tumor suppressor genes regularly seen deleted in bladder cancer to model transformation of naÃ¯ve to malignant cells.

Comments for the author

Taken together, this is a concise and very solid work that demonstrates a robust method for the generation of large deletions in human cells that promises to be realizable with mostly basic methods in molecular cell biology. Even though the idea is not new, the clean and robust workflow

© 2023. Published by The Company of Biologists under the terms of the Creative Commons Attribution License (https://creativecommons.org/licenses/by/4.0/).

that is shown with unimpeachable documentation justifies, from my point of view, publication of the story essentially in its present form in JCS.

I still suggest some issues that should be addressed prior to publication:

The HSP90 loading control in Fig. 1e seems reduced in ko cells as compared to controls. To avoid misunderstandings, this blot should be replaced / redone.

Plots in Figs. 1 g and S1d seem to include negative values, which should be clarified / corrected.

A(nother) centriolar protein should be analyzed in CEP128 kos cells to show that centrioles are still intact.

Cells having lost CEP128 seem to grow happily (?). The authors should at least comment/discuss (better have evidence) if this is an expected behavior and what it may mean for centrosome function.

Throughout the text, the authors mention at several points that the documented workflow comprises high-throughput analysis (e.g. 2x in the abstract). I think this is at least misleading. Even though the method may have to potential for a high throughput approach, this is not addressed in this paper. I suggest toning down to s.th like “the method may be suitable for high throughput approaches due to…”.

First revision

Author response to reviewers' comments

Reviewer #1 (Comments for the Author):
Major issues 1.
The data in Figure 3 contrast with those in Figure 2, in that a 1000 kb deletion was achieved with an efficiency similar to that seen with a 100 kb (albeit with a low frequency for the 590 kb deletion). If this is to be ascribed to the individual gRNAs, a direct demonstration of different cutting efficiency should be provided. Otherwise, it is not clear what can be concluded, beyond the basic feasibility of large deletions using the authors’ protocol.

We thank this reviewer for this comment. To address this reviewer’s request, we have performed the T7E1 mismatch cleavage assay to analyze the cutting efficiency of individual gRNAs used in the Figure 3 (new data in Fig. S4E). For the 590-kb deletion, we re-designed and tested two new gRNA versions (new 590- kb#1 and #2) because the cutting frequencies of the previous 590-kb gRNAs in the original manuscript were below the detection limit of this approach. Genomic DNA was extracted from the cells electroporated with Cas9 proteins and individual gRNAs. The target genomic region was amplified by PCR and the PCR product was then subjected to the T7E1 assay. As shown in the new Fig. S4E, a higher amount of digested DNA heteroduplexes was detected for those gRNAs, which performed efficient deletion in the CRISPR-del assay (new data in Fig. 3F). In contrast, the sgRNAs that resulted in low deletion rates could be also correlated to stronger bands of intact PCR product in the T7E1 assay, which indicates lack of editing. The difference in the size of digested bands is due to the position of the individual cutting sites in the PCR products. These data indicate that the low efficiency of some gRNAs observed in the CRISPR-del assay is due to their low cutting efficiency. We have added the new data and modified the result section accordingly (page 9, lines 18-24 in the revised manuscript).

2. The use of a pool in the HNRNPA1 experiment is problematic for several reasons. First, the binary readout means that the deletion of one tagged allele in a bi- allelically labelled cell will not be detected by the FACS assay. This issue may confound the quantitation. Second, the relative percentage of cells with a mono- and bi- allelic mNG tag may vary between experiments, which may also impact on the outcomes. Third, a putative mNG-negative population may proliferate differently after transfection, possibly leading to other confounding factors. These experiments should be repeated with a clonal starting population.

© 2023. Published by The Company of Biologists under the terms of the Creative Commons Attribution License (https://creativecommons.org/licenses/by/4.0/).

We thank the reviewer for pointing this out. We have extensively addressed this reviewer's valid points with a new set of experiments. In response to the request, we established a mono-allelic *HNRNPA1-mNG* knock-in clone of RPE1 cell (new data in Fig.S4A,B,C) and repeated all the experiments from the original version of Fig.3 using this clone. Overall, the outcome of the new experiments is similar to the original data using the *HNRNPA1-mNG* knock-in cell pool, strengthening the validity of our conclusion. We have replaced the previous data with the new data using the mono-allelic knock-in clone in Fig.3 (new data in Fig.3B,C,D,E,F) and modified the result section accordingly (page 8 and 9 in the revised manuscript).

3. With respect to the HNRNPA1 locus experiment, a further issue is whether the disruption of any of the protein coding genes in which the guides are sited may be toxic to cells, individually or in combination, and thus affect the outcome of the editing experiments. The 2.4% of mNG negative cells with the 1000 kb deletion into the MYG1 locus might argue against that, but information about the genetic interactions between the other loci is limited. The authors should be cautious about this aspect.

We thank the reviewer for this important comment. As this reviewer mentioned, the successful deletion of the 1000 kb indicates that the disruption of the coding genes within this genomic region would not be toxic to RPE1 cells in the time frame of this experiment (FACS analysis was performed 8 days after electroporation). Although no obvious difference in cell proliferation between conditions was observed in this experiment, the possibilities pointed out by the reviewer cannot be ignored. Therefore, we have commented on this in the revised manuscript, noting that the efficiency of CRISPR-del may be under- or over-estimated in this experiment (page 9, lines 28-30 in the revised manuscript).

4. Was a percentage editing determined for the experiment in Fig. 1? It would be useful to extend the curve information in Figure 2c, if this could be included.

We thank this reviewer for this constructive comment. Unfortunately, we had not determined the percentage of the deletion efficiency for the experiment in Fig.1.

5. The PCR experiments in Figs. 1d, 2d, 4b, 4c, S1a, S2b and S3f should include a positive control.

We have added a positive control for genomic PCR data in Figs. 1D, 2D, 4C, S1A, S2B, S3B, S4B and S4F in the revised manuscript, as requested by the reviewer.

6. Details of the RPE1 cell line used in this study and its source should be provided.

We have provided information on the source of the RPE1 cell line in the Material and Method section (page 13, line 3 in the revised manuscript).

Minor points 7.
In terms of individual clone handling, how are individual/ selected clones of interest woken up from the 96 well dish?

For thawing frozen cells, we describe the following protocol in the Material and Method section: "The frozen 96-well plates were submerged in a water bath at 37°C. After thawing, the clones of interest were transferred into tubes containing fresh medium. After centrifugation and subsequent removal of supernatant, the cell pellet was resuspended with fresh medium and transferred into a new 96-well plates for culture." (page 14, lines 12-16 in the revised manuscript).

8. With a binary outcome (mNG positive/ negative), it is not helpful to present the data in Figure 3 as stacked columns. A bar graph should indicate only one of the values and the y-axis could be adjusted to extend only to 30%, which would be much clearer.

We have modified the bar graphs of the new data in Fig.3D and F to make them much clearer, as suggested by the reviewer.

© 2023. Published by The Company of Biologists under the terms of the Creative Commons Attribution License (https://creativecommons.org/licenses/by/4.0/).

Reviewer #2 (Comments for the Author):
They made six or seven points which are critical for generation of complete gene knockout cells with high efficiency. However, it would not be easy for an average laboratory to install all the points indicated in the manuscript. Some points should be more critical for deletion efficiency than the others. Therefore, this reviewer suggests to perform control experiments to determine the following points.

- The authors used RPE1 cells. They may try additional cell lines to make sure that their method also guarantees a high deletion efficiency in diverse cell types.

We thank this reviewer for this constructive comment. As requested by this reviewer, we have now also applied the CRISPR-del pipeline to HCT116, which is a human colorectal carcinoma cell line having a near-diploid karyotype. We designed and performed a strategy to introduce a 27-kb deletion into the locus of *Kinesin Light Chain 1* (*KLC1*) gene in HCT116 cells as well as RPE1 cells by using our optimized CRISPR-del (new schematic representation in Fig.S2A). Genomic PCR analysis of more than 100 clones revealed successful generation of mono- and bi-allelic deletion clones for both cell lines (new data in Fig.S4B). The deletion efficiencies for RPE1 and HCT116 cell lines were 21.6 % and 39.0 %, respectively (new data in Fig. S2D). The deletion efficiency achieved by the CRISPR-del pipeline varies depending on the cell line, target site and gRNAs. Nevertheless, the newly added data demonstrates that high knockout rates can be reached in diverse cell types using our CRISPR-del method. We have added these new data in the revised manuscript and modified the result section accordingly (page 6, line 29 to page 7, line 5 in the revised manuscript).

- It would be useful if the authors compare transfection efficiency of electroporation and other transfection methods.

We thank the reviewer for this comment. In addition to electroporation, we now also showed data for lipofection-mediated CRISPR-RNP delivery using Lipofectamine CRISPRMAX (Thermo Fisher Scientific) for the 27-kb deletion of the *KLC1* gene in RPE1 cells. This delivery method succeeded in deleting the target region, but with a considerably lower efficiency than that of the electroporation method (new data in Fig. S2B). We have added this data to the revised manuscript and modified the result section accordingly (page 6, lines 32- 34 in the revised manuscript).

- The authors should explain how to determine target sequences of a specific gene.

We design gRNA sequences using available CRISPR gRNA design tools, CRISPOR (Concordet and Haeussler, 2018) and Custom Alt-R® CRISPR-Cas9 guide RNA (Integrated DNA Technologies). We have added this information to the Material and Methods section (page 13, lines 11-12 in the revised manuscript).

- The bi-allelic deletions of CEP128 were much less efficient than the expectation from the mono-allelic deletions. The authors interpreted that the bi-allelic knockout of CEP128 reduces the proliferative ability of the cells. This reviewer suggests to perform the same experiment with another genes whose deletions do not affect cell survival and proliferation.

We thank the reviewer for this important comment. To address this reviewer's request, we targeted *Kinesin Light Chain 1* (KLC1) gene as mentioned above. Based on the available literature, KLC1 is not considered to be involved in cell proliferation, so we introduced a large deletion into this gene by our CRISPR-del method (new schematic representation in Fig.S2A). We found 32 (20.9%) and 17 (11.1%) clones harboring mono- and bi-allelic deletions, respectively, among 152 surviving clones for RPE1 cell line (new data in Fig.S2D). For HCT116 cell line, the frequency of the mono- and bi-allelic deletions were calculated as 53.3% (56/105) and 12.4% (13/105), respectively. Although the ratio of mono- and bi- allelic deletions of *KLC1* varies between the two cell lines, it is much higher than that of *CEP128* in RPE1 cells (69.8% and 3.2% for mono- and bi-allelic 20-kb deletions in Fig.2B). Therefore, these results are consistent with our interpretation that bi-allelic knockout of *CEP128* gene reduces the proliferative ability of the cells. We have added these data to the revised manuscript and modified the result section accordingly (page 6, line 29 to page 7, line 5, and page 7, lines 32 to page8, lines 2 in the revised manuscript).

© 2023. Published by The Company of Biologists under the terms of the Creative Commons Attribution License (https://creativecommons.org/licenses/by/4.0/).

Reviewer #3 (Comments for the Author):
Taken together, this is a concise and very solid work that demonstrates a robust method for the generation of large deletions in human cells that promises to be realizable with mostly basic methods in molecular cell biology. Even though the idea is not new, the clean and robust workflow that is shown with unimpeachable documentation justifies, from my point of view, publication of the story essentially in its present form in JCS.

I still suggest some issues that should be addressed prior to publication:

The HSP90 loading control in Fig. 1e seems reduced in ko cells as compared to controls. To avoid misunderstandings, this blot should be replaced / redone.

We thank this reviewer for this comment. We have repeated the Western blot and confirmed that there was no difference in the expression levels of HSP90 between the conditions. We have replaced the blot image in Fig. 1E.

Plots in Figs. 1 g and S1d seem to include negative values, which should be clarified / corrected.

We thank the reviewer for this comment. The negative values come from the step of subtracting the cytoplasmic background signal in the quantification. Antibody staining gives uneven background in immunofluorescence analysis. A negative value means that the CEP128 signal at centrosomes is below the level of the background due to the absence of centrosomal CEP128 in the CEP128 deleted clone. To avoid any misunderstandings of this data, we have added a note in Material and Methods section explaining how we performed the signal quantification for immunofluorescence data (page 16, lines 19-24 in the revised manuscript).

A(nother) centriolar protein should be analyzed in CEP128 kos cells to show that centrioles are still intact. Cells having lost CEP128 seem to grow happily (?). The authors should at least comment/discuss (better have evidence) if this is an expected behavior and what it may mean for centrosome function.
We thank the reviewer for this suggestion. We have performed an immunofluorescence analysis using an antibody against centrin, which is a well- defined centriole marker. Similar to WT cells, centrin signals were observed in the CEP128 deleted clones (new data in Fig. 1C), indicating the presence of centrioles in the clones. We have added this data to the revised manuscript (new data in Fig.1SC) and modified the result section accordingly (page 6, line 26, and page8, line 7 in the revised manuscript).
CEP128 is known to be a component of an accessory structure of the mother centriole called the sub-distal appendage (Mazo et al. 2016), but its function in cell growth has not been reported. As mentioned in the comment for reviewer #2, our data suggest that CEP128 may play a role in cell proliferation, but is not necessarily essential. It would help to elucidate a novel function of CEP128 in the future.

Throughout the text, the authors mention at several points that the documented workflow comprises high- throughput analysis (e.g. 2x in the abstract). I think this is at least misleading. Even though the method may have to potential for a high throughput approach, this is not addressed in this paper. I suggest toning down to s.th like “the method may be suitable for high throughput approaches due to...”.

We thank this reviewer for this helpful suggestion. In response to this suggestion, we have toned down the claim of “a high-throughput approach” in the text, e.g., to “an efficient and practical approach” in the abstract (page 2, line 20 in the revised manuscript).

© 2023. Published by The Company of Biologists under the terms of the Creative Commons Attribution License (https://creativecommons.org/licenses/by/4.0/).

Second decision letter

MS ID#: JOCES/2022/260000

MS TITLE: A CRISPR-del-based pipeline for complete gene knockout in human diploid cells

AUTHORS: Takuma Komori, Shoji Hata, Akira Mabuchi, Mariya Genova, Tomoki Harada, Masamitsu Fukuyama, Takumi Chinen, and Daiju Kitagawa
ARTICLE TYPE: Tools and Resources

I am happy to tell you that your manuscript has been accepted for publication in Journal of Cell Science, pending standard ethics checks.

Reviewer 1

Advance summary and potential significance to field

The revision of the manuscript on CRISPR-mediated deletions by Komori, Kitagawa and colleagues has addressed all the points that were raised in the initial review, both generalising their findings and providing significant additional detail. This work now presents a useful and convincing study of technical aspects relating to large gene deletions, which will be of technical interest for researchers using CRISPR-mediated reverse genetics in a wide variety of fields.

The authors discuss the elements of the well-established used of CRISPR-del that have been optimised in this study and, although the improvements are individually incremental, the presentation of these elements together will be of benefit to readers of the Journal of Cell Science. Therefore, I recommend the publication of this submission.

Comments for the author

As it is likely that the Materials and Methods section of this paper will be used for reference, it would be appropriate to fix some outstanding minor points (although I do not consider that any further review is needed):
1. Company names (Nacalai Tesque, Abcam, etc.) should be capitalised throughout the Materials and Methods, to avoid confusion.
2. The area used for fluorescence intensity measurement in the microscopy analyses should be specified.
3. It is not clear whether the specified A302-479A antibody is an anti-centrin reagent (my search has this as a CEP152 antibody from Bethyl). This should be checked/ corrected.
4. The reference list is duplicated; this should be tidied up.
5. The description of siRNA-mediated gene knockdown should be checked in the Materials and Methods (my version of the PDF for review has some squares, which may confuse readers).

Reviewer 2

Advance summary and potential significance to field

Comments for the author

The authors fulfilled all the points that had been raised.

Reviewer 3

Advance summary and potential significance to field

Komori et al. hand in a revised version of the ms on a "A CRISPR-del-based pipeline for complete gene knockout in human diploid cells".

© 2023. Published by The Company of Biologists under the terms of the Creative Commons Attribution License (https://creativecommons.org/licenses/by/4.0/).

Comments for the author

I appreciate the careful consideration of the suggestions that I made but even more that the authors have been taking care of suggestion of the other two reviewers. Taken together, the revision has further optimized the work in a way that it now makes an excellent contribution to JCS. Having said this, I fully support publication of the manuscript in its present form.

© 2023. Published by The Company of Biologists under the terms of the Creative Commons Attribution License (https://creativecommons.org/licenses/by/4.0/).
